# Association between time spent in the Russian Federation and late presentation for HIV among Tajikistani migrants

**DOI:** 10.1186/s12889-020-09434-6

**Published:** 2020-09-10

**Authors:** Daniel J. Bromberg, Mary M. Tate, Kamiar Alaei, Saifuddin Karimov, Dilshod Saidi, Arash Alaei

**Affiliations:** 1 Department of Social and Behavioral Sciences, Yale School of Public Health, Yale University, New Haven, CT USA; 2 Center for Interdisciplinary Research on AIDS (CIRA), Yale University, New Haven, CT USA; 3 Department of Epidemiology of Microbial Diseases, Yale School of Public Health, Yale University, New Haven, CT USA; 4 Institute for International Health and Education, Albany, NY USA; 5 Department of Health Sciences, California State University, Long Beach, USA; 6 Republican AIDS Center, Tajikistan Ministry of Health, Dushanbe, Tajikistan

**Keywords:** Eastern Europe and Central Asia, HIV, Migration

## Abstract

**Background:**

Between 700 thousand and 1.2 million citizens of Tajikistan currently live in the Russian Federation, one of the only countries where the HIV epidemic continues to worsen. Given the previously reported barriers to healthcare access for migrants to the Russian Federation, and the rapidly expanding HIV epidemic in Eastern Europe and Central Asia, this present study set out to determine whether these barriers impact late presentation with HIV among Tajikistani migrants upon their return to Tajikistan.

**Method:**

This study uses data from the Tajikistan Ministry of Health surveillance system (2006 – 2019). At time of diagnosis, patients are interviewed by staff of AIDS centers, and doctors complete routine intake forms and complete medical exams. Descriptive characteristics of migrants with HIV who had lived in the Russian Federation (n=503) were calculated and compared with those of non-migrants with HIV (n=9519). Missing data were imputed using multiple imputation (predictive means matching, logistic regression imputation, and polytomous regression imputation). Two logistic models were created to model the probability of late presentation for HIV. The first model shows unadjusted associations between predictor variables and late presentation for HIV. The second model shows multivariable associations between significant study variables identified in the univariate model, and late presentation.

**Results:**

Compared to non-migrants, migrants with HIV are more likely to be from Gorno-Badakhshan region, are less likely to use illicit drugs, and are more likely to have purchased the services of sex workers. The unadjusted logistic model found that for every year spent in the Russian Federation, the risk of late presentation for a Tajikistani migrant with HIV increases by 4.0% (95% CI: 0.3-7.7). The multivariate model showed that when age, sex, and region of origin are held constant, the risk of late presentation for a Tajikistani migrant with HIV increases by 4.0% (95% CI: 0.1-7.8) for each year spent in the Russian Federation.

**Conclusion:**

The results of this paper suggest that if the Russian Federation were to loosen its restrictions on HIV care for foreign nationals, it might improve the treatment outcomes of migrant laborers. As this analysis is only correlational in nature, further research is needed to explicate the causal pathways of the associations found in the present analysis.

## Introduction

For the past decade, the Russian labor force has been progressively shrinking. As a result, the Russian labor supply has not been able to meet the labor demand required to maintain economic growth [1]. Economic analysis of data from the Russian Federal State Statistics Service predicts that the Russian Federation’s current labor shortages will only increase if drastic policy measures are not taken to increase the labor force [2]. To maintain economic productivity, Russia relies on migration from former Soviet Union states, most notably from Central Asia. Central Asian labor migrants act as a failsafe for the Russian economy in the context of a declining native-born working-age population, national financial instability, and international sanctions.

Migration has been a defining aspect of Central Asian social and economic structures since the fall of the Soviet Union. The Tajikistani civil war which accompanied the republic’s independence in the 1990s instigated the first major flow of forced migrants from Central Asia in the post-Soviet period. The country never recovered economically, and today relies on the exportation of its labor force for a large portion of its economic output. In 2013, Tajikistan was the country with the world’s greatest proportion of its GDP (41.7%) coming from remittances [3].

Media reports claim that, as of 2015, the Russian Federation has implemented stricter requirements for work permits, with special relevance for Tajik migrants. Tajik migrants must now pay relatively high fees (reportedly between $200 and $500 USD), prove sufficient proficiency in the Russian language, pass certain exams, and submit complicated paperwork in order to receive a valid work permit. As Tajik migrants can enter Russia without a visa, strict labor registration requirements arguably encourage illegal labor [4]. Without medical insurance or the right to care, as well as the fear of legal recourse if found to be working unregistered, it is very possible that Tajik migrants in the Russian Federation forgo much-needed HIV testing and treatment. Moreover, the Russian Federation restricts entry to the country based on HIV-status, presumably to protect the country from an exacerbated HIV epidemic, mirroring the former policy of the United States. A qualitative study conducted among migrants in Moscow revealed that, because of the Russian Federation’s mandatory reporting of confirmed cases of HIV, at least some migrants refused HIV-tests out of fear of expulsion or refusal of re-entry into the Russian Federation [5].

Due to the ban on migrants with HIV in the country, migrants are generally unable to access HIV treatment or care through the Russian healthcare system. Although few alternative HIV treatment options exist, there are NGOs such as Moscow Based group “Shagi Foundation”, which works with HIV positive migrants to find solutions to any barriers they may face including the procurement of antiretroviral therapy through informal networks [6, 7]. There are also informal community-based healthcare providers, such as the so-called “Kyrgyz clinics”, which specifically target Central Asian populations and provide them with a range of health care services and assistance with navigating the formal medical system in Russia [8].

Migrants must prove an HIV-negative status in order to obtain an annual work permit in Russia [9]. As citizens of the Commonwealth of Independent States, Tajik migrants can enter Russia without a visa and apply for a work permit after arrival to Russia [10]. Therefore, migrants who were infected pre-migration could enter and work in Russia without a work permit [7]. Working in Russia without a legal work permit, however, confines migrants to jobs within the black-market and bars them from accessing healthcare and other resources in the country.

In Tajikistan, HIV testing is readily available and prioritizes labor migrants [11]. After pregnant women, migrants (defined as all citizens who stay abroad for three months or more) constitute the largest portion of people tested [12]. As of 2018, returning migrants are required to undergo HIV testing upon re-entry to Tajikistan [13]. Recent qualitative data suggest that migrants who are diagnosed with HIV in Russia often choose to remain in the country until advanced symptoms force them to return to Tajikistan to seek adequate medical treatment and free ART, which is otherwise unavailable in the Russian Federation [7, 14].

Russia has higher HIV prevalence than Tajikistan among its adult population (> 1% of the population vs 0.3, respectively) [11, 15]. Previous research also suggests that Central Asian migrants are more likely to engage in HIV risk behavior in Russia than at home [16]. An elevated incidence of HIV has also been recorded among wives of Tajik migrants following their husbands’ return home [17]. The higher prevalence of HIV in Russia combined with the high-risk that Tajik migrants face in contracting the disease implies that migrants are more likely to become infected in Russia than in Tajikistan.

If Tajikistani migrants do not receive testing or treatment for HIV infections in the Russian Federation, they may face the health consequences associated with late presentation. Late presentation with HIV and subsequent late initiation into antiretroviral therapy increases the risk of HIV-related complications, reduces life expectancy and quality, and reduces the net benefits of ART generally. On the other hand, those virally suppressed on ART have virtually no risk of transmitting the virus to HIV-negative people [18]. Importantly for the public health of the Russian Federation, not initiating migrants in antiretroviral therapy may increase the risk of HIV infection being spread even beyond migrant populations. The heightened vulnerability of migrants in Russia has the potential to spill-over beyond the boundaries of the Russian Federation and puts local population in home countries at heightened risk of an exacerbated HIV epidemic.

Additionally, studies conducted among migrants with HIV to European countries, suggests that a substantial proportion of migrants acquired HIV following migration to Europe [19, 20]. A cross-sectional study by Alvarez-del Arco, et al. showed that the proportion of post-migration acquisition of HIV among migrants to nine European countries was 63% overall, but as high as 79% for people who inject drugs (PWID) and 71% for men who have sex with men (MSM). The factors associated with post-migration acquisition among migrants to the nine European countries included length of stay with longer duration of residence tied to a higher probability of HIV post-migration acquisition [19]. Similar histories of migration pathways, social hardship, and healthcare barriers may indicate an epidemiological pattern of post-migration HIV acquisition applicable to Central Asia and the Russian Federation. Given the previously reported barriers to healthcare access for migrants in to the Russian Federation, and the rapidly expanding HIV epidemic in Eastern Europe and Central Asia, this present study set out to determine the characteristics of HIV-positive migrants

## Methods

This study uses data from the Tajikistan Ministry of Health surveillance system. The dataset contains all new known HIV diagnoses between 2006 and 2019. Time of diagnosis is generally coincident with return to Tajikistan; in this sample, 94% of patients with HIV were diagnosed for the first time after their return to Tajikistan; and the median time from return to diagnosis was 6 months. Six percent of the sample reported being diagnosed for the first time before their return to Tajikistan; the median time from diagnosis to return for these patients was also 6 months. At time of diagnosis, patients are interviewed by staff of AIDS centers, complete routine intake forms and complete medical exam and related forms by doctors. As part of these routine intake forms, migration information from the patients’ passports (including date of exit and re-entry to Tajikistan) are collected. These data give supplementary information on each patient’s sexual history, alcohol and drug use, and migration history, in addition to other factors. Mean CD4 count is also taken at this time.

**Fig. 1:**
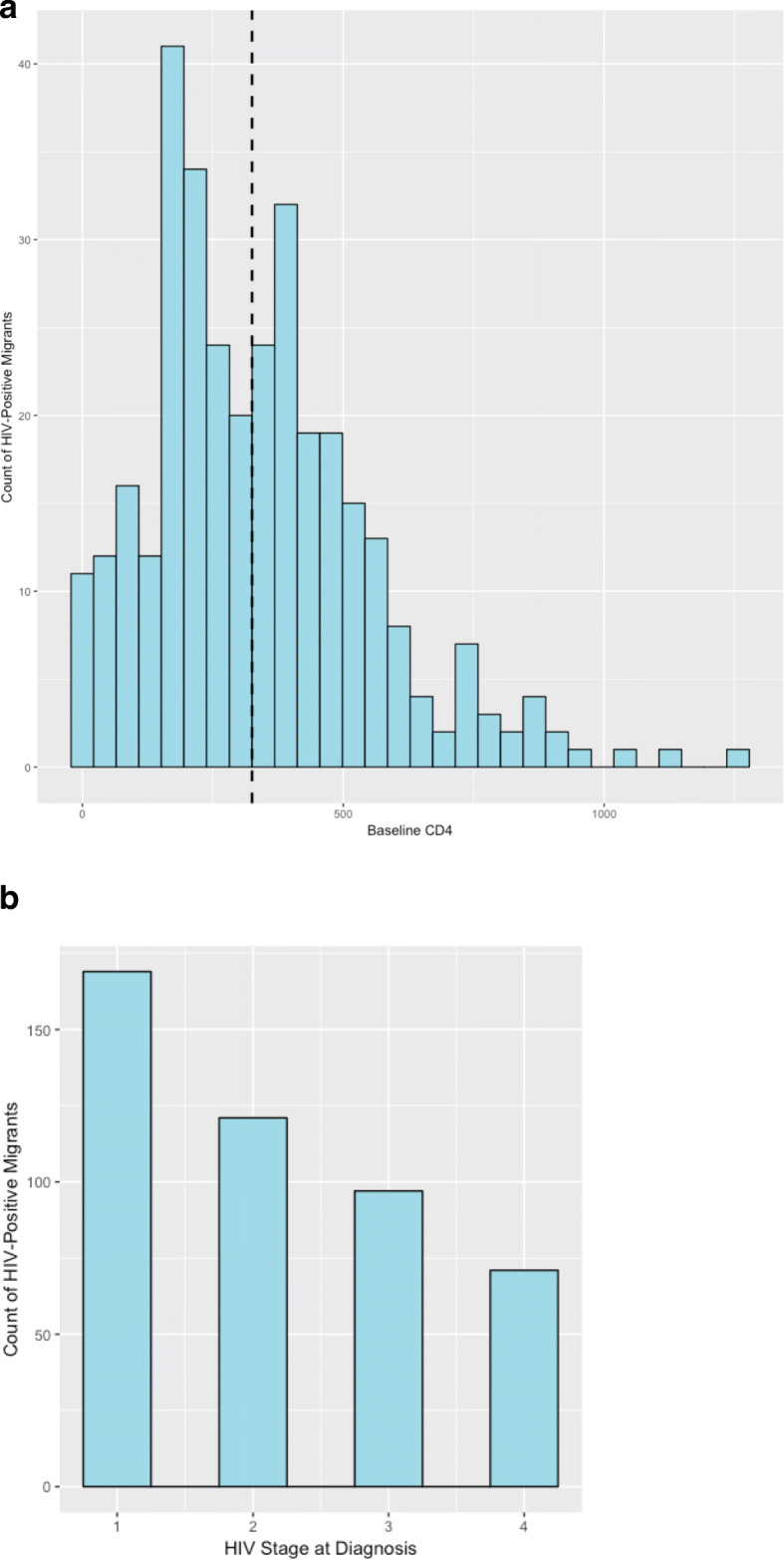
**a** Distribution of Baseline CD4 Counts for Returned Migrants with HIV (Median CD4 = 325). **b** Distribution of Clinical Stage of HIV at Diagnosis Legend: HIV Stages at Diagnosis (1 = asymptomatic [37%], 2 = Mild Symptoms [26%], 3 = Advanced Symptoms [21%], 4 = AIDS [16%])

Late presentation was defined as any person who had a CD4 count lower than 200 cells/mL. People with HIV who had been migrants to the Russian Federation and for whom complete migration data were available (*n* = 503) were included in final data analysis (Fig. 1). People with HIV who had not been migrants outside of Tajikistan were included as controls (*n* = 9519).

Descriptive statistics were calculated for migrants with HIV who had returned from the Russian Federation, as well as for non-migrants. Variables included in tabulation of descriptive statistics are demographic information, measures of HIV progression, HIV transmission route, HIV risk behavior, and non-HIV co-infections. To determine if there were statistically significant differences between migrants with HIV and non-migrants with HIV, *p*-values were calculated. For continuous variables that are normally distributed, these *p*-values correspond to a t-test. For continuous variables that are not normally distributed, MannWhitney U tests were performed. For categorical variables, p-values correspond to a Pearson Chi-Square test (Table 1).

**Table 1:** Characteristics of People Living with HIV in Tajikistan, Stratified by Migrants and Non-Migrants

	Non-Migrants ***N = 9519***	Migrants ***N = 503***	***P***-Value*
**Region:**			< 0.001
Badakhshan	398 (4.18%)	98 (19.5%)	
DRS	1939 (20.4%)	103 (20.5%)	
Dushanbe	2430 (25.5%)	40 (7.95%)	
Khatlon	2667 (28.0%)	129 (25.6%)	
Sughd	2085 (21.9%)	133 (26.4%)	
**Age (mean)**	37.2 (13.4)	39.6 (8.30)	< 0.001
**Age (Categorical):**			< 0.001
< 18	1120 (11.8%)	0 (0.00%)	
18–24	247 (2.59%)	10 (1.99%)	
25–34	1991 (20.9%)	152 (30.2%)	
35–44	3324 (34.9%)	201 (40.0%)	
45–55	2175 (22.8%)	117 (23.3%)	
55<	662 (6.95%)	23 (4.57%)	
**Percent Female**	40% (0.49)	16% (0.37)	< 0.001
**Transmission Route:**			
Heterosexual	4834 (50.8%)	406 (80.7%)	
Injection drug use	3219 (33.8%)	92 (18.3%)	
Male-to-Male Sexual	36 (0.38%)	4 (0.80%)	
**Relationship Status:**			< 0.001
Divorced	1048 (11.8%)	56 (11.5%)	
Married	4583 (51.7%)	344 (70.9%)	
Unmarried	2761 (31.1%)	60 (12.4%)	
Widowed	380 (4.29%)	17 (3.51%)	
**Education:**			. < 0.001
Elementary	180 (2.12%)	6 (1.25%)	
High school	6498 (76.4%)	353 (73.5%)	
None	815 (9.58%)	7 (1.46%)	
Specialized high school	416 (4.89%)	80 (16.7%)	
Unfinished high school	304 (3.57%)	5 (1.04%)	
Unfinished University	80 (0.94%)	8 (1.67%)	
University Degree	214 (2.52%)	21 (4.38%)	
**Employment:**			< 0.001 .
Employed	555 (5.83%)	86 (17.1%)	
Incarcerated	207 (2.17%)	1 (0.20%)	
Military	18 (0.19%)	0 (0.00%)	
None	8300 (87.2%)	413 (82.1%)	
Other	87 (0.91%)	1 (0.20%)	
Retired	26 (0.27%)	1 (0.20%)	
Student	308 (3.24%)	0 (0.00%)	
Student (higher education)	17 (0.18%)	1 (0.20%)	
**Ever had Male-to-Male Sexual Contact**:			0.688
No	5903 (98.4%)	415 (98.8%)	
Yes	94 (1.57%)	5 (1.19%)	
**Ever used Illicit Drugs:**			< 0.001
No	5942 (64.7%)	400 (79.7%)	
Yes	3240 (35.3%)	102 (20.3%)	
**Baseline_CD4 (mean)**	390 (305)	340 (213)	< 0.001
**Late Presentation (CD4 < 200) at Diagnosis**:			0.505
No	3900 (73.2%)	234 (71.3%)	
Yes	1429 (26.8%)	94 (28.7%)	
**Number of Sexual Partners in the Last 12 Months (mean)**	2.29 (3.11)	3.05 (2.87)	< 0.001
**Percent AntiHCV Positive**	27% (0.44)	30% (0.46)	0.393
**Percent TB Positive (Syndromic)**	2% (0.14)	2% (0.15)	0.811
**Percent HBsAg Positive**	7% (0.26)	6% (0.25)	0.615
**Ever Purchases Services of a Sex Worker:**			< 0.001
No	4392 (68.3%)	247 (50.7%)	
Yes	1827 (28.4%)	209 (42.9%)	

*For continuous variables that are normally distributed, a t-test is performed to calculate *p*-values. For continuous variables that are not normally distributed, MannWhitney U is performed. For categorical variables, p-values correspond to a Pearson Chi-Square test

Missing data were imputed using a multiple imputation model, in accordance with established multiple imputation guidelines [21, 22]. Before imputation, data were checked to determine if variables were missing completely at random (MCAR), missing at random (MCAR), missing at random (MAR). For variables that had missing data (Baseline CD4 Count [Late Presentation], Education, Relationship Status, and risk behavior variables), missingness analysis using the *finalfit* package in R was conducted [23]. The *finalfit* packages cross-tabulates independent and dependent variables, stratified by missingness (missing and not-missing), and then performs Chi-square tests to determine whether there are statistically significant differences among strata.

Using the *mice* package in R [24], missing data were imputed for all migrants with HIV. Data were checked for normality, and numeric variables were imputed using predictive mean matching (e.g., number of sexual partners in the last 12 months), binary data (e.g., late presentation) were imputed using logistic regression imputation, and unordered categorical data (e.g. education) were imputed using polytomous regression imputation. Using these methods, 50 imputed datasets were created. The models assumed MCAR where the missingness analysis showed no statistically significant difference in missingness by strata, and MAR for all other variables.

Two logistic models were created to model the probability of late presentation for each of the imputed data sets (*n* = 50). The estimates for each dataset were then pooled using Rubin’s rule [25] to determine the final model parameters. The first model shows unadjusted associations between predictor variables and late presentation for HIV. The second model is a multivariable logistic model which shows multivariable association between only those study variables found to be influential in the univariate model, as well as region of origin and gender, and late presentation. Data analysis was conducted using R Studio Version 4.0 [26].

## Results

Five hundred and three returned migrants with HIV were included in this study. Most of the sample is male (86%), and roughly representative of the five regions of Tajikistan, although relatively fewer of the migrants are from the capital, Dushanbe (8%). Compared with non-migrants with HIV, they are significantly more likely to be from Gorno-Badakhshan region, are less likely to get HIV from injection drug use or have ever used illicit drugs, are more likely to have purchased the services of sex workers, and have more sexual partners on average. About a fifth of the migrants reported to be people who inject drugs (PWID) (Table 1).

Missingness analysis showed that late presentation information is more likely to be missing among migrants from Gorno-Badakhshan region, migrants between the ages of 45 and 54, and among those whose HIV transmission was through injection drug use. The results of the missingness analysis for the variable Late Presentation are presented in (Table 2). For all other variables, missingness was less than 5% of the set and all variables were MCAR.

**Table 2:** Missingness analysis for the Late Presentation variable. Categories for which the Late Presentation variable is not missing completely at random (MCAR) are in bold. *P*-values correspond to a chi-square test

Category	Category	Not Missing	Missing	P-value
Age				0.017
	18–24	9 (2.7)	1 (0.6)	
	25–34	110 (33.5)	42 (24.0)	
	**35–44**	**127 (38.7)**	**74 (42.3)**	
	**45–54**	**65 (19.8)**	**52 (29.7)**	
	> 55	17 (5.2)	6 (3.4)	
Region				< 0.0001
	**Badakhshan**	**46 (14.0)**	**52 (29.7)**	
	DIRS	74 (22.6)	29 (16.6)	
	Dushanbe	26 (7.9)	14 (8.0)	
	Khatlon	88 (26.8)	41 (23.4)	
	Sughd	94 (28.7)	39 (22.3)	
Sex	Male	272 (82.9)	150 (85.7)	0.495
	Female	56 (17.1)	25 (14.3)	
Education	Elementary	3 (1.0)	3 (1.8)	0.326
	High school	238 (76.0)	115 (68.9)	
	None	6 (1.9)	1 (0.6)	
	Specialized high school	45 (14.4)|	35 (21.0)	
	Unfinished high school	2 (0.6)	3 (1.8)	
	Unfinished University	5 (1.6)	3 (1.8)	
	University Degree	14 (4.5)	7 (4.2)	
Relationship Status				0.010
	Single	13 (4.2)	12 (6.9)	
	Divorced	38 (12.2)	18 (10.4)	
	Married	230 (73.7)	114 (65.9)	
	Other	5 (1.6)	1 (0.6)	
	**Unmarried (Not Single)**	**14 (4.5)**	**21 (12.1)**	
	Widowed	12 (3.8	5 (2.9)	
Transmission Route				
	Heterosexual	282 (86.0)	124 (70.9)	< 0.001
	Injection Drug Use	**41 (12.5)**	**51 (29.1)**	
	Male-to-Male Sexual	4 (1.2)	0 (0.0)	
Employment				0.132
	Employed	65 (19.8)	21 (12.0)|	
	Unemployed	260 (79.3)	153 (87.4)	
Ever had Male-to-Male Sexual Contact				0.235
	No	267 (98.2)	148 (100.0)	
	Yes	5 (1.8)|	0 (0.0)	
Ever used Illict Drugs				
	No	279 (85.3)	121 (69.1)	
	Yes	48 (14.7)	54 (30.9)	
Ever Purchased Services of Sex Worker				0.259
	No	154 (48.4)	93 (55.0)	
	Yes	145 (45.6)	64 (37.9)	

Two logistic models were created to model the probability of late presentation. The unadjusted logistic model found that for every year spent in the Russian Federation, the risk of late presentation for a Tajikistani migrant with HIV increases by 4.0% (95% CI: 0.3–7.7) (Table 3). The multivariable model showed that when holding age, region of origin, and gender constant, the risk of late presentation for an HIV-positive Tajikistani migrant increases by 4.0% (95% CI: 0.1–7.8) for each year spent in the Russian Federation (Table 4). Common HIV-risk behaviors like number of sexual partners in the last 12 months, drug or alcohol use, or sex with commercial sex workers, were not found to be statistically significant predictors of late presentation for HIV in either iteration of the logistic model.

**Table 3:** Unadjusted associations between study variables and probability of late presentation for HIV (*n* = 503)

Characteristic	OR (95% Confidence)	P-value
**Region of Origin**		
Badakhshan	1.00	
Districts of Republican Subordination	0.9 (0.4–1.8)	0.8
Dushanbe	0.9 (0.4–2.4)	0.9
Khatlon	0.9 (0.4–1.8)	0.7
Sughd	0.7 (0.4–1.5)	0.4
**Current Age (years)**		
18–24	1.00	
25–34	2.1 (0.2–17.5)	0.5
35–44	3.5 (0.4–29.1)	0.2
45–54	4.5 (0.5–38.3)	0.2
**> 55**	**15.9 (1.6–160.2)**	0.02**
As continuous*	**1.06 (1.03–1.09)**	0.0001***
**Time Spent in Russia**		
Less than 5 years	1.00	
5–15 years	1.5 (0.9–2.4)	0.1
**15 years or more**	**1.9 (1.0–3.7)**	0.05**
As continuous*	**1.04 (1.003–1.077)**	0.03***
**Sex**		
Male	1.00	
Female	0.7 (0.3–1.3)	0.3
**Education**		
Elementary	1.00	
High school	1.2 (0.1–11.1)	0.9
None	0.6 (0.0–11.0)	0.8
Specialized High School	1.0 (0.1–10.3)	0.8
Unfinished High School	1.4 (0.06–34.5)	1.0
Unfinished University	1.6 (0.1–27.1)	0.8
University Degree	1.7 (0.1–21.1)	0.7
**Employment Status**		
Employed	1.00	
Unemployed	1.3 (0.7–2.4)	0.4
**Relationship Status**		
Single	1.00	
Divorced	2.9 (0.5–17.7)	0.3
Married	2.4 (0.4–13.4)	0.3
Widowed	4.4 (0.6–32.7)	0.1
**Transmission Route**		
Heterosexual	1.00	
Injection Drug Use	1.4 (0.7–2.5)	0.3
Male-to-Male Sexual	0.0 (undefined)	0.1
**Number of Sexual Partners in Last 12 months**		
As continuous*	1.0 (0.9–1.1)	0.8
**Risk Behavior & Misc.**		
Ever used illicit drugs		
No	1.00	
Yes	0.3 (−0.3–0.9)	0.3
Ever had Male-to-Male Sexual Contact		
No	1.00	
Yes	−13.6 (− 1207.8–1180.7)	1.0
Ever Purchased Services of Sex Worker		
No	1.00	
Yes	0.2 (−0.3–0.6)	0.4

**Table 4:** Multivariable logistic regression model of factors associated with late presentation for HIV (N = 503)

Characteristic	Adjusted OR (95% CI)	p
**Time Spent in Russia**	1.04 (1.001–1.078)	0.04*
**Age**		
18–24	1.00	
25–34	2.2 (0.3–19.3)	0.5
35–44	3.7 (0.4–31.9)	0.2
45–54	4.6 (0.5–40.1)	0.2
**> 55**	**18.3 (1.7–195.7)**	0.02**
**Sex**		
Male	1.00	
Female	0.7 (0.3–1.4)	0.3
**Region of Origin**		
Badakhshan	1.00	
DRS	1.0 (0.5–2.2)	1.0
Dushanbe	0.9 (0.4–2.9)	0.9
Khatlon	0.9 (0.5–2.2)	1.0
Sughd	0.7 (0.4–1.8)	0.6

## Discussion

This study established that the only statistically significant variable in predicting late presentation for HIV among returned Tajik migrant workers, other than age, is their time spent in the Russian Federation. For every year spent in the Russian Federation, the risk of late presentation for HIV increases by 4.0%. Interestingly, risk behavior was not found to predict late presentation. The implication of this finding is that structural factors in the Russian Federation, and not migrants’ behavior, is the most important factor affecting HIV treatment outcomes. With the average person in the present sample spending a median 7 years in the Russian Federation, this risk has significant implications for individual and public health, in both Tajikistan and the Russian Federation, as well as Eastern Europe and Centrals Asia more broadly. The findings of this study support the authors’ hypothesis that Russia’s policies that deny or discourage HIV testing and treatment are more important in affecting the HIV epidemic among migrant workers than are behavioral factors of the migrants themselves.

In the early days of the epidemic, HIV initially spread along migration routes [27–29]. A systematic review by Weine, et al. showed that prolonged or frequent absences were among the most frequently cited determinants of HIV risk in studies involving labor migrants [30]. Indeed, length of time spent away from home has been shown to be a strong predictor of HIV positive status in several international settings [31–33]. A previous study based on large surveys of male migrants in the Russian Federation has found that difficulty in accessing healthcare services and limited health education contributed to Tajik migrants’ HIV vulnerability [17]. Other studies have shown high levels of HIV-related risk behavior among Tajik migrants to Russia [16, 34], including sex with commercial sex workers, sex with multiple partners, and excessive alcohol use. Unlike these past studies, the present findings found no association between these risk behaviors and HIV treatment outcomes.

The domestic economies of the Russian Federation and Tajikistan are reliant to different degrees on migrant labor. As of 2013, between 700 thousand and 1.2 million Tajik workers were living and working in Russia, with remittances constituting 41% of the Tajikistan’s GDP [3, 35]. It is in the economic interest of both the Tajikistani and Russian governments to ensure the health and wellbeing of Tajik migrant workers. As barriers to legal residency status are ample [36, 37] the Russian government should take appropriate legal action to ensure the ability of Tajik workers to obtain legal work permits. A legal working status allows migrant laborers to have greater access to the state healthcare system and has also been shown to reduce occurrences of racial discrimination against Central Asian migrants, which often act as a barrier in accessing healthcare (ibid). Preventative HIV measures would also need to be better resourced and integrated into the Russian healthcare system. These measures should include HIV education, promotion of condom use, regular HIV testing and access to clean needles for people who inject drugs. In improving HIV treatment outcomes and preventing late presentation, the Russian government should also consider the difficulty in obtaining antiretroviral therapy for migrant workers, especially those without a legal working status. It is also important to recognize the experience of racism and ethnoracial harassment in the Russian Federation [10]. Instances of informal racism transcend any legal and social protections imposed on migrant laborers by the Russian state. Although informal racism has been recorded to occur less in instances of legal working status [10, 36], Central Asian labor migrants continue to report a high occurrence of racialized harassment and prejudice in and outside their work environment (ibid). Socio-structural barriers, created by discrimination, act as deterrents in accessing preventative HIV measures and are linked to increased HIV risk among migrant laborers. The Russian government will have to address the broader cultural, social and political factors associated with informal racism to remove the socio-structural barriers related to increased HIV risk [36].

While the present study used data from a large and comprehensive dataset and most potential confounders were accounted for, regression models are limited in their ability to prove causality. Information on risk behavior were reported directly from medical reports rather than patients. The reliability of medical reports may have been compromised, particularly in reporting of injection drug use (IDU), as high levels of hepatitis C infection suggest that the level of IDU is higher than reported.

Missingness analysis found some bias in the record keeping of late presentation at diagnosis. Notably, records of late presentation were more likely to be missing among PWID, middle-age individuals, and people from Gorno-Badakhshan. Gorno-Badakhshan is the poorest and most remote region of Tajikistan, with a predominantly Ismai’ili Shi’a population that often faces ostracism from other sectors of the country. It is reasonable that the HIV centers in this region are less equipped to keep ideal records of HIV cases. As drug use is heavily stigmatized, PWID are perhaps less likely to be linked to care following diagnosis and may be less likely to consent to CD4 count screening. While it is unlikely that these omissions systematically biased the results of the present paper, the reporting flaws point to opportunities for HIV record keeping improvement in Tajikistan.

Due to heavy stigma, MSM are also likely underreported in the present sample. Recent literature has shown that stigma, fear of disclosure, and limited access to healthcare has prevented MSM from accessing HIV testing in Tajikistan [38]. As such, to conduct robust research on HIV among Tajik MSM and reduce bias in the future, multi-level anti-stigma interventions [39] should be enacted in the country. The timing of diagnosis following arrival (a left-skewed distribution with a median of 6 months) suggests selective return-migration of migrants experiencing HIV symptoms. The selective return of less healthy migrants, a so-called “salmon bias”, may partially explain why returned migrants are more likely to be late presenters for HIV when compared to non-migrants [40–42]. For these reasons, the authors of the present study urge for more research to explore the mechanisms underlying the associations presented in the present study. Future research can use causal inference and qualitative methodologies to supplement the present findings.

## Conclusion

The government of Tajikistan has no policy of deporting people with HIV, and the Republican AIDS Center provides testing and treatment to all people, including migrants, regardless of their nationality. The results of this paper suggest that if the Russian Federation were to adopt a similar policy, it might improve the treatment outcomes of migrant laborers. Further research is needed to explicate the causal pathways of the associations found in the present analysis.

## Data Availability

The data that support the findings of this study are the property of the Ministry of Health of Tajikistan and are not publicly available. Dr. S Karimov and D. Saidi co-authors are staff of the Tajikistan Ministry of Health and had permission to access to the data and analyze it in collaboration with other authors.
